# The effectiveness of exercise intervention for academic achievement, cognitive function, and physical health among children in Mongolia: a cluster RCT study protocol

**DOI:** 10.1186/s12889-019-6986-8

**Published:** 2019-06-06

**Authors:** Kenji Takehara, Togoobaatar Ganchimeg, Akihito Kikuchi, Lkagvasuren Gundegmaa, Lkagvasuren Altantsetseg, Ai Aoki, Takemune Fukuie, Kazuya Suwabe, Shagdar Bat-Erdene, Masashi Mikami, Rintaro Mori, Hideaki Soya

**Affiliations:** 1 Department of Health Policy, National Center for Child Health and Development, 2-10-1, Okura, Setagaya, Tokyo, 157-8535 Japan; 2 Global Health Nursing, Faculty of Medicine, University of Tsukuba, 1-1-1, Tennodai, Tsukuba, Ibaraki, 305-8577 Japan; 3 Laboratory of Exercise Biochemistry and Neuroendocrinology, Faculty of Health and Sport Sciences, University of Tsukuba, Tsukuba, 305-8574 Ibaraki Japan; 4 Division of Sport Neuroscience, Advanced Research Initiative for Human High Performacnce (ARIHHP), Faculty of Health and Sport Sciences, University of Tsukuba, Tsukuba, 305-8574 Ibaraki Japan; 5 Mongolian National Institute of Physical Education, P.O.Box-224, Ikh Toiruu-49, Sukhbaatar district, Ulaanbaatar, Mongolia; 6 Department of Neuropsychiatry, Graduate School of Medicine, The University of Tokyo, 7-3-1 Hongo, Bunkyo, Tokyo, 113-8655 Japan; 7 Division of Biostatistics, National Center for Child Health and Development, 2-10-1, Okura, Setagaya, Tokyo, 157-8535 Japan

**Keywords:** RCT, Physical activity, Exercise, Academic achievement, Physical fitness, Cognitive function, Children

## Abstract

**Background:**

Many studies have demonstrated positive effects of physical activity on children’s health such as improved cardiorespiratory function and decreased obesity. Physical activity has also been found to have positive effects on academic achievement and cognitive function. However, there are few high quality RCT studies on this topic at present and the findings remain controversial.

**Methods:**

This protocol describes cluster randomized controlled trials assessing the impact of school-based exercise intervention among children in Mongolia. The intervention consists of 3-min sessions of high intensity interval training combined with music implemented two times a week at school during study periods. The participants are children in the fourth grade in public elementary schools in the Sukhbaatar district in Ulaanbaatar, Mongolia. The participants are cluster randomized by school and allocated either to the intervention or control group. The primary outcome is academic achievement. Secondary outcomes are obesity/overweight, physical fitness function, lifestyle, mental health, and cognitive function.

**Discussion:**

This cluster-RCT is designed and implemented to assess the effectiveness of exercise intervention on academic achievement, cognitive function, and physical and mental health among school-age children in Mongolia. This study will provide evidence to promote physical activities among children in low- and middle- income countries.

**Trial registration:**

UMIN: UMIN000031062. Registered on 1st February 2018.

## Background

Physical inactivity is becoming an urgent health issue. Although physical inactivity is highest in developed countries, it is also on the rise among middle-income earners in developing countries including their children [1]; the WHO reported that 81% of adolescents aged 11–17 years worldwide had insufficient levels of physical activity. [1]

Previous studies demonstrated a strong relationship between physical inactivity and non-communicable diseases such as obesity, hypertension, diabetes, elevated blood lipids, heart attack, heart failure, stroke, and breast and colon cancers [1, 2]. Physical activity in childhood is crucial for the healthy development of musculoskeletal tissue, the cardiovascular system, neuromuscular awareness, and prevention of obesity and diabetes. [1, 3] Moreover, recent studies have suggested that physical activity contributes to improving brain function and mental health. [4–8] Several ongoing studies are focusing on the positive effects of physical activity in broader areas such as academic achievement, cognitive function, mental health, and social skills. [3, 6, 9–15]

Academic achievement is a composite measure of many different factors from the developmental to the psychosocial. Several mechanisms thought to be instrumental in improving academic achievement have been reported from the neurobiological, psychological, and physical perspectives. Neurobiologically, exercise induces increased cerebral blood flow, brain-derived neurotrophic factors, neuroplasticity, and changes in neurotransmitters such as serotonin, noradrenalin, and dopamine. [4, 5] For example, the acquisition of motor skills was shown to improve neuroplasticity and cognitive function, [5] and improvements in executive function, memory, and attention have been reported in connection with increased physical activity. [16–20] The executive function is considered to be especially important for predicting children’s academic achievement. [21] Psychologically, exercise is thought to improve mood, lessen anxiety, and boost self-perception/self-esteem, all of which contribute to better academic achievement. [3, 11, 22] Numerous studies have demonstrated the positive effects of exercise on academic achievement and cognitive function, but most were small randomized controlled trials (RCTs), and some were not intervention studies. [10, 16, 17, 23–26] To date there are very few, large, population-based RCT examining the effect of exercise in children. A small number of meta-analyses and systematic reviews have been done but have demonstrated only small positive effects due to exercise intervention. [8, 12, 27] A Cochrane systematic review recommended investigating academic achievement and brain function in obesity-prevention studies because only few RCT addressing these issue exist [28] and the current evidence is meager. High-quality RCT are needed especially in developing countries as most of the previous intervention studies were done in developed countries like the US, Canada, and Australia.

As studies of children in the developed world have shown, physical activity deficits among children not only result in increased obesity and poorer athletic ability but may also have adverse effects on academic achievement and brain function. Similar trends are now being seen among children in emerging nations. Mongolia is one of these emerging nations now facing an epidemiological transition from infectious diseases to non-communicable and lifestyle-related health issues. In Mongolia, the physical activity deficit among children has become a serious problem in recent years especially in Ulaanbaatar, the capital city, where the shortage of outdoor play spaces caused by rapid urbanization, the spread of television and video games, and severe winter weather, have reduced the physical activity levels of children. In Ulaanbaatar, the average temperature is below freezing for more than five months per year. This fact has contributed greatly to increasing child obesity. In Mongolia, previous studies showed that the rate of overweight and obesity was 10.6 and 1.5%, respectively, among children aged 12–17. [29] Physical inactivity and obesity cause childhood health problems including diabetes mellitus, hypertension, hyperlipidemia, etc. Moreover, the adverse effects of exercise deficits are thought to have non-physical consequences including poor academic achievement and cognitive development. These health problems are common to middle-income earners worldwide, including those in developing countries like Mongolia.

The primary objective of our study is to investigate the effectiveness of exercise intervention on academic achievement among children using a large cluster RCT. The secondary objective is to investigate the effectiveness of exercise intervention on reducing the prevalence of obesity and overweight and on improving physical fitness performance, lifestyle, mental health, and cognitive function. The executive function was chosen as the target cognitive function based on the hypothesis that this function can best predict academic achievement.

## Methods/design

This study will be a collaborative effort by the National Center for Child Health and Development, Mongolian National Institute of Physical Education (MNIPE), and the University of Tsukuba. This study is a cluster-RCT using school as a cluster. A pilot study has already been done to confirm the feasibility and logistics of the planned intervention program and the assessment of the subjects’ physical fitness and cognitive function at one school in the study area.

### The study setting

This study will be conducted in the Sukhbaatar district of Ulaanbaatar, Mongolia and will recruit 10 public schools out of 11 public schools in the district. The population of this district in 2017 was 136,569 or roughly 10% of the population of Ulaanbaatar. [30]

One school which is not recruited for this study participated in the pilot study. Ten schools will later be allocated to the intervention or control group. Six, three, and two schools are located in urban or residential, *ger* (Mongolian traditional dwelling), and mixed residential areas, respectively. Each school has a total of 108 to 307 children in four to nine fourth-grade classes.

### Participants

Participants are children in the fourth grade in public elementary schools in the study area. The inclusion criteria are: [1] attendance at a public school in the Sukhbaatar district; [2] written consent from parents or guardians; and [3] age-appropriate literacy in Mongolian. The exclusion criteria are: [1] comorbidities or contraindications prohibiting participation in an exercise program and [2] enrollment in a special needs program.

### Intervention

The exercise program for the intervention group will be implemented two times per week at school in twice-weekly physical education classes required by the educational curriculum. The exercise program combines high intensity interval training and music. The exercise program will consist of four segments with different target activities and short rest intervals between segment. The target activities of each segment are warm-up, agility and high-speed movements, side-steps, and jumps. The program requires 3 min. In the first phase of intervention, the participants will practice each segment one by one to synchronize their movements with the music. Including stretching and explanation of the program before the exercise program, it will take about 20 min. The first phase will last for six weeks. Subsequently, in the second phase of the intervention, the participants will perform all the segments sequentially. Including stretching, it will take 10 min. The second phase will last for four weeks.

The program, based on high intensity interval training (HIIT), will consist of high intensity phases involving steps, jumps, and squats alternating with intervals of rest. HIIT has been found to improve physical endurance effectively. Some studies using small samples have also reported that HIIT improved not only physical endurance but also cognitive function more efficiently to compare with moderate-intensity endurance training. [31–34] This exercise program uses music, sound effects and verbal instructions in Mongolian to help children, especially those who are poor at physical activities, to acquire the movement easily. For this purpose, the tempo of music is set at 105-140 bpm. The music is also chosen carefully to amuse and motivate children. [35, 36] This program does not use self-paced or acquired movements such as walking. Through this program, the participants will aim to acquire a variety of motor skills, which is thought to correlate with improvements in cognitive function. This intervention will be administered by university students at MNIPE working as research assistants (RA). The RA will undergo a five-day course covering the aims of the program, appropriate practices, safety measures including ensuring the health and well-being of the participants, and training in implementing the exercise regimen. The RA will be instructed using standardized materials at the course and receive a few days additional course to improve the quality of intervention program during the intervention period. This program will be implemented in a space available at each school, such as the gymnasium or conference room. Although practice is recommended at least two times per week, each school will decide the actual frequency. There are no criteria for discontinuing participation or modifying the allocation. During the study period, MNIPE researchers will visit schools in the intervention group on a regular basis and supervise implementation of the program. In the control arm, the participants will attend the usual physical education class only.

### Primary outcome

The primary outcome of academic achievement will be assessed by the participants’ performance on the examination in 2018, which consists of a mathematics and Mongolian language portion. The participants’ score on the test for May 2017 at the end of third grade will be used as the baseline data.

### Secondary outcomes

The proportion of obesity and overweight, and the physical fitness function, lifestyle, and mental health, and cognitive function measures between November and December 2018 will be the secondary outcomes. All measures will be obtained in between February and April in 2018 as baseline data.

The researchers and RAs will obtain physical measurements (height and body weight) and physical fitness function test data to establish the baseline in February and at the end of study. The body mass index (BMI) will be calculated, and the proportion of participants classified as obese or overweight using the WHO/CDC criteria will be calculated. Physical fitness function will be assessed by participants’ performance on a 20-m shuttle run and side-to-side jumps.

Participants’ parents or guardians will answer a self-administered questionnaire on lifestyle covering their children’s sleeping hours, exercise habits, time spent viewing television or using video games, children’s household tasks, and their mental and physical health. Children’s physical health condition will be assessed using a five-point Likert scale. The Strengths and Difficulties Questionnaire (SDQ), which has already translated into Mongolian and back-translated, [37] will be used to assess the mental health of the participants. The cut-off values on the SDQ published in the literature and available on the official website for ‘normal,’ ‘borderline,’ and ‘abnormal’ will be adopted. [38]

To assess cognitive function, the Flanker test, a representative cognitive function test specifically assessing inhibitory control of the executive function, will be used. [39] The Flanker test will be administered using a specially-designed app for use on a tablet at school under the instruction of the researchers. The response time and correct rate for 50 tasks, including congruent and incongruent tasks, will be measured. Before the actual test, every student will do a practice test consisting of ten tasks once or twice.

**Fig. 1:**
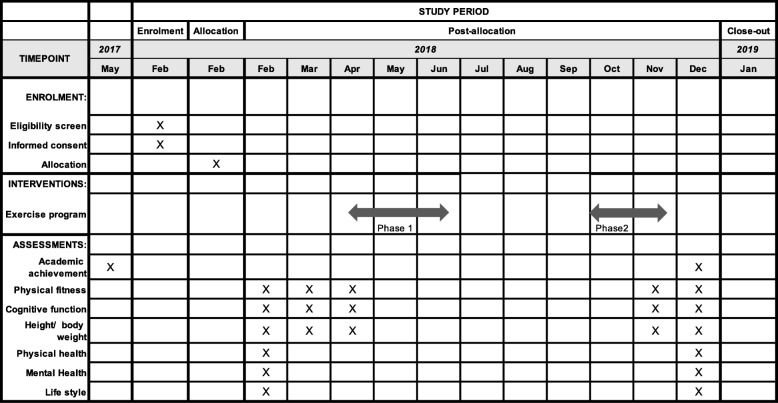
The schedule of enrolment, interventions and assessments

The intervention will begin in April 2018. Randomized allocation is done in February 2018 and baseline data collection will be done from February to April 2018. The intervention period will be interrupted by summer vacation between the end of June and the beginning of September 2018. The intervention period before summer vacation is the first phase of the intervention and after summer vacation is the second phase of the intervention. Post-intervention data will be collected between November and December 2018. (Fig. 1).

### Sample size

Some 2445 children are enrolled in fourth grade at public elementary schools in the Sukhbaatar district of Ulaanbaatar, Mongolia. 2337 children are enrolled in the ten schools that participate in this study. All the eligible children will be recruited.

This study is a longitudinal cluster randomized trial which has three-level cluster units: the school, individual, and measurement time point. Additionally, the number of students at each school differs. To calculate the statistical power, we implemented a simulation study extending the method of a previous study to adjust for unbalanced data. [40] We assumed the standardized difference of the primary outcome to be equal to 0.2, and the dropout rate to be equal to 5%. Since the statistical power of the longitudinal cluster randomized trial depends on the correlation between measurement time points for the same individual, we assumed values of 0.4, 0.5 or 0.6. Bonferroni correction was used to adjust for the multiplicity of statistical testing calculated for each site (Urban/*ger*/mixed residential). Table 1 shows the statistical power and alpha error for each site. We also simulated the disjunctive alpha error and power. In all assumption settings, the disjunctive alpha error is kept nearly equal to 0.05, and the statistical power of the urban site exceeds 0.8. Additionally, under conditions in which the correlation equals 0.6, the statistical power exceeds 0.8 for both sites. The simulation was done 5000 times using the mixed procedure with SAS Software 9.4 (SAS Inc., Cary, NC, USA).

**Table 1:** Simulation results of statistical power

Correlation	Disjunctive: 5000 simulations Standardized difference = 0.2					
	Alpha error			Power		
	Total	Urban	*Ger* or mixed residential	Total	Urban	*Ger* or mixed residential
0.4	0.050	0.027	0.024	0.957	0.871	0.642
0.5	0.047	0.023	0.024	0.977	0.920	0.719
0.6	0.047	0.023	0.024	0.992	0.967	0.814

### Allocation

We will perform stratified block randomization to allocate ten schools into the intervention and control arms. These schools will be stratified into the urban area group (6 schools) and the *ger* or mixed residential area group (4 schools) according to the location of the school. To ensure a balance between the two study arms, we will order the schools by the number of fourth grade students to form blocks for randomization in each residential group. Within the blocks, the schools will be randomly allocated to the intervention or control arm. To allocate the schools, arbitrary numbers given by the representative of each school will be used. The arbitrary numbers will be processed by a program on R statistical software, version 3.4.3 (The R foundation for statistical computing platform) which is developed by a researcher. Each school will be randomly allocated to intervention or control group based on the output of the R program.

The allocation will be conducted and announced to the representative of each school at a kickoff meeting.

### Blinding

According to the nature of the intervention, the participants and outcome raters, participants’ parents or guardians answering the questionnaires, and researchers taking the anthropometric and physical fitness function measurements will not be blinded to the allocation. However, the chief data analyst will be blinded to the allocation.

All data collected in this study will be entered in duplicate and managed using Microsoft Excel. The data file will be secured with a password, and access to information will be limited to designated members such as the researchers and RA. Paper-based documents such as consent forms, questionnaire response sheets, and forms for recording the physical fitness function and anthropometric measurements will be stored at a research institute in Ulaanbaatar. The data from the Flanker test will be stored on tablets and the internet server.

### Statistical methods

#### Analysis of the primary outcome

The baseline data of the intervention and control groups will be compared. The chi-squared test will be used for binary variables and the t-test will be used for continuous variables. To avoid overestimating the impact of the intervention, an intention-to-treat approach will be adopted to evaluate the impact on the primary and secondary outcomes. In this study, an individual’s data will be analyzed in the originally allocated arm even if the individual moves to a school in another area during study period.

To examine the effect of the intervention while taking the hierarchical structure into consideration, we will use a linear mixed effect model. In this model, the fixed effects are the group (intervention/control), site (Urban/ *ger* or mixed residential), and measurement time point with the following two interaction terms: the interaction between group and measurement time point, and the interaction between the group, measurement time point, and site. The random effects are school and individual. The degrees of freedom will be estimated using Satterthwaite approximation. For the primary analysis, we will compare the participants’ performance on the national year-end examination before and after the intervention using least squares means estimated by the interaction term of the group, measurement time point, and site. The Bonferroni method will be used as a multiplicity adjustment method. A significance level of 0.025 will be set for a two-sided test, and the 95% confidence interval will be calculated.

#### Analysis of secondary outcomes

In the secondary analysis, the same analytical method will be used as in the primary analysis for each test score (mathematics and language) and the continuous outcomes of BMI, sleeping hours, 20m shuttle run, each element of the Flanker test, side-to-side movement, and each SDQ score for mental health. We will use mixed-effects logistic regression for binary data. The covariate of this model is also the same as in the primary analysis. Although the frequency of exercise and playing outside hours constitute ordered data, they will be converted into binary data using a cutoff in the secondary analysis. The cutoff of the frequency of exercise will be between “1–2 days” and “3–4 days”, playing outside hours will be between “30 min-59 min” and “60 min-119 min”. Since the analysis of the secondary outcome is the exploratory evaluation, we will not adjust for the multiplicity of statistical testing.

In the analysis of cognitive function using the Flanker test, participants who fail to reach a correct rate of 50% will be excluded on the assumption that they do not understand the test.

In this study, any imputation will not be performed to missing data due to lost participants or imperfect data collection. The data will be analyzed using STATA V.13.1 (STATA/IC 13.1 for Mac, STATA Corp, Lakeway Drive College Station, Texas), SAS Software 9.4 (SAS Inc., Cary, NC, USA), and SPSS statistics V.19.0 (IBM SPSS Statistics for Windows, IBM Corp, Armonk, New York).

#### Subgroup analysis

The effect of the intervention will be further analyzed using four subgroup analyses. The first subgroup analysis will focus on the location of the schools (urban and non-urban areas), the second on gender, and the third on baseline physical fitness performance using the median score on a 20m shuttle run. The fourth subgroup analysis will focus on baseline academic achievement using the total score on the national year-end examination in May 2017 and the median score. The fifth subgroup analysis will be done after excluding participants whose correct rate on the Flanker test is below 80% at either the baseline or endline surveys.

### Other additional analysis

An additional analysis will be done after adjusting the sample size of each cluster to a certain number. The number will be decided according to the number of participants in the smallest cluster.

## Discussion

This paper is a study protocol for a cluster-RCT to assess the effectiveness of exercise intervention on academic achievement, cognitive function, and physical and mental health among school-age children in Mongolia. The intervention program combines exercise and music and was designed for use with an educational curriculum already filled with many subjects and activities.

### Monitoring

Although this study is an intervention trial, the intervention is not considered to be invasive. Thus, no data monitoring committee or auditing will be needed. To ensure compliance with the intervention program, an RA will be assigned to each primary school in the intervention arm to take charge of monitoring and will be responsible for biweekly reports on the progress of the study including the frequency of the intervention program at the respective school. Researchers both in Mongolia and Japan will discuss the progress of the study and issues to be addressed via Skype on a biweekly basis in addition to frequent e-mail communication.

### Risks, burdens, and benefits

The intervention program will consist of high intensity interval training. However, the physical stresses induced by the intervention, such as an increase in respiratory rate, heart rate, and sweating, are assumed to fall within the range of normal physical activity and to require a brief recovery time, given appropriate rest and hydration. In the pilot study, we confirmed that the rating of perceived exertion (RPE) using the Borg Rating of Perceived Exertion Scale normalized after the intervention. [41] The Ethical Guidelines for Medical and Health Research Involving Human Subjects in Japan have also assessed the intervention planned for this study, which is very similar to the physical fitness function test used in Japanese schools, to be non-invasive. Based on these assessments, the exercise regimen planned for this study is not considered to be harmful to the participants.

Prior to the commencement of the study, local researchers and teachers will be instructed in appropriate and safe practices for avoiding the risk of medical emergencies during the implementation of the intervention. The burden of responding to the questionnaire will fall to the parents or guardians of the participants, while the participants will have the burden of undergoing the cognitive function test. This part of the study will provide no direct benefit to the participants.

### Modification and inquiry

Any modification or inquiry regarding the study will be communicated through the Department of Health Policy at the National Center for Child Health and Development. During the study period, a research office will be opened at the MNIPE. Inquiries from Mongolia will be gathered at the research office and processed by the Department of Health Policy at the National Center for Child Health and Development.

### Confidentiality

The personal data including school information, class, name, social security number, etc. contained in the consent forms will be stored at the MNIPE. All data will be anonymized using a research ID number when they are entered into a computer for storage. The research ID and corresponding table will be stored at the MNIPE.

### Post trial care

If the study demonstrates no negative effects, school teachers and children in the control groups will be taught the exercise program by the RAs after the study.

### Dissemination policy

The results of this study will be disseminated to professionals and organizations in education and shared publicly throughout Mongolia. The study findings will also be disseminated at peer reviewed scientific journals and presented at international conferences and conferences in Japan, Mongolia, and other countries.

## Abbreviations

BMI: Body mass index
CDC: Centers for Disease Control and Prevention
HIIT: High intensity interval training
MNIPE: Mongolian National Institute for Physical Education
RA: Research assistant
RCT: Randomized controlled trial
RPE: Rating of perceived exertion
SDQ: The Strength and Difficulties Questionnaire
WHO: World Health Organization
